# A Practical Comparison of Short‐ and Long‐Read Metabarcoding Sequencing: Challenges and Solutions for Plastid Read Removal and Microbial Community Exploration of Seaweed Samples

**DOI:** 10.1111/1755-0998.14129

**Published:** 2025-06-04

**Authors:** Coralie Rousseau, Nicolas Henry, Sylvie Rousvoal, Gwenn Tanguy, Erwan Legeay, Catherine Leblanc, Simon M. Dittami

**Affiliations:** ^1^ Integrative Biology of Marine Models (LBI2M, UMR 8227), Station Biologique de Roscoff Sorbonne Université, CNRS Roscoff France; ^2^ ABiMS, FR2424, Station Biologique de Roscoff Sorbonne Université, CNRS Roscoff France; ^3^ Genomer Platform, FR2424, Station Biologique de Roscoff Sorbonne Université, CNRS Roscoff France; ^4^ Adaptation and Diversity in the Marine Environment (UMR 7144), Station Biologique de Roscoff Sorbonne Université, CNRS Roscoff France

**Keywords:** adaptative sampling, bacteria, fungi, Illumina, macroalgae, Oxford Nanopore Technology

## Abstract

Short‐read metabarcoding analysis is the gold standard for accessing partial 16S and ITS genes with high read quality. With the advent of long‐read sequencing, the amplification of full‐length target genes is possible, but with low read accuracy. Moreover, 16S rRNA gene amplification in seaweed results in a large proportion of plastid reads, which are directly or indirectly derived from cyanobacteria. Primers designed not to amplify plastid sequences are available for short‐read sequencing, while Oxford Nanopore Technology (ONT) offers adaptive sampling, a unique way to remove reads in real time. In this study, we compare three options to address the issue of plastid reads: deleting plastid reads with adaptive sampling, using optimised primers with Illumina MiSeq technology, and sequencing large numbers of reads with Illumina NovaSeq technology with universal primers. We show that adaptive sampling using the default settings of the MinKNOW software was ineffective for plastid depletion. NovaSeq sequencing with universal primers stood out with its deep coverage, low error rate, and ability to include both eukaryotes and bacteria in the same sequencing run, but it had limitations regarding the identification of fungi. The ONT sequencing helped us explore the fungal diversity and allowed for the retrieval of taxonomic information for genera poorly represented in the sequence databases. We also demonstrated with a mock community that the SAMBA workflow provided more accurate taxonomic assignment at the bacterial genus level than the IDTAXA and KRAKEN2 pipelines, but many false positives were generated at the species level.

## 1 Introduction

The study of microbial communities by high‐throughput marker gene sequencing (i.e., metabarcoding) is a popular method to identify a large proportion of cultivable and non‐cultivable species (Ruppert et al. 2019). Illumina is one of the most commonly chosen technologies for such studies and is carried out using either the MiSeq or the NovaSeq sequencers. Both allow for remarkable microbial characterisation thanks to high sequencing depth and highly accurate reads, but there are some differences: the MiSeq produces paired reads of 2 × 300 bp length with a capacity of 15 Gb per V3 flowcell, whereas an SP flowcell on a NovaSeq 6000 produces 2 × 250 bp with up to 400 Gb output (Ravi et al. 2018; Modi et al. 2021). The NovaSeq generally yields forward and reverse high‐quality reads, whereas the reverse reads of the MiSeq are of lower quality (Han et al. 2024). In both cases, the taxonomic assignment is based on short variable regions with a size, generally up to about 400 bp, and the resolution of this method is, in most cases, insufficient to discriminate sequences at the species level. Moreover, the study of fungal diversity could be challenging with short‐read sequencing, as the ITS region (i.e., Internal Transcribed Spacer) exhibits intraspecific length variations ranging from 300 to 1300 bp (Schoch et al. 2014). These difficulties are even more important in understudied groups with incomplete reference databases, such as marine fungi (Hassett et al. 2019).

Compared to Illumina, Oxford Nanopore Technology (ONT), a third‐generation sequencing technology, allows the sequencing of longer stretches of DNA, including full‐length 16S and ITS rRNA genes for prokaryotic and eukaryotic species (~1500–1600 bp) in real time (Deamer et al. 2016; Santos et al. 2020). The disadvantage of this technology compared to Illumina is the high error rate (Jain et al. 2017). Comparative analysis between Illumina and ONT instruments, across a range of sample types—including mouse, human, bivalve, soil, and lanternfish—often reports only minor differences (Shin et al. 2016; Winand et al. 2019; Heikema et al. 2020; Matsuo et al. 2021; Low et al. 2021; Egeter et al. 2022; Stevens et al. 2023; van der Reis et al. 2023).

Brown macroalgae host a wide variety of microscopic organisms, including bacterial, archaeal, eukaryotic, and viral communities (Egan et al. 2013). Many brown seaweeds (i.e., macroalgae) depend on their microbial partners for normal growth, development, nutrient supply, and protection from colonisation and predation (Wahl et al. 2012; Egan et al. 2013; Singh and Reddy 2014). These host–microbe associations form a functional, sometimes co‐dependent, entity known as a holobiont (Egan et al. 2013; van der Loos et al. 2019; Dittami et al. 2021; Saha et al. 2024; Marzinelli et al. 2024). While some information on the seaweed‐associated epibacterial community is available (Martin et al. 2015; Weigel and Pfister 2019; Parrot et al. 2019; Paix et al. 2021; Quigley et al. 2020; Burgunter‐Delamare et al. 2023; Brunet et al. 2024), very few studies focus on the endophytic community as well as other members of the holobiont. Nevertheless, seaweeds are colonised by eukaryotic partners. A clear example of this is the presence of the endophytic fungus *Mycophycias ascophylli* in the brown alga *Ascophyllum nodosum* , which may influence host development and protect against desiccation (Garbary and Gautam 1989; Garbary and MacDonald 1995; Garbary and London 1995; Garbary et al. 2015). Reliable and exhaustive surveys of all the members of the holobiont, including those within the host, are crucial for understanding brown seaweed holobionts.

To date, the exploration of macroalgae‐associated microbiomes has mainly been performed using short‐read (Illumina) metabarcoding (Rousseau et al. 2024) and only in a few cases long reads, for example, to characterise the microbiome associated with the green alga *Ulva* (van der Loos et al. 2022). One of the difficulties in using long‐read sequencing with algal or, more generally, plant samples is related to the high proportion of plastid reads recovered when using standard 16S primers, which can represent > 85% of the total reads in a sequencing run (Thomas et al. 2020). This is due to the cyanobacterial origin of the plastids (Jensen and Leister 2014). A simple option to overcome this problem is to increase the sequencing coverage. Alternatively, Thomas et al. (2020) have designed primers to specifically avoid plastid amplification for the MiSeq sequencer (Illumina). However, these primers result in the loss of some bacterial phyla, and the amplicon length (Table 1) is optimised for short‐read sequencing. For ONT long‐read sequencing, adaptive sampling (i.e., Readfish, Loose et al. 2016) may be a solution to the issue of plastid sequences, as suggested previously (van der Loos et al. 2021).

**TABLE 1:** Summary of sequencing methods, primer choices, and PCR cycling conditions. 16S rRNA and ITS regions were chosen as targets for the bacterial and fungal kingdoms, respectively. Mean amplicon sizes were calculated after read quality filtering.

Sequencing	DNA region	Mean amplicon size (bp)	Kingdom targeted	Forward primer	Reverse primer	PCR cycling	Reference
Illumina MiSeq (2 × 300 pb)	V3‐V4 16S rRNA	380	Bacteria, archaea	S‐B‐bact‐0341‐b‐S‐17F: CCTACGGGNGGCWGCAG	799F_rc: CMGGGTATCTAATCCKGTT	– 95°C, 2 min – 30 denaturation cycles (95°C, 1 min), primer hybridization (53°C, 30 s), primer extension (72°C, 2 min) – Final extension step 72°C, 5 min	Thomas et al. (2020)
Illumina NovaSeq (2 × 250 pb)	V4‐V5 16S rRNA	375	Bacteria, archaea, eukaryotes	515F: GTGYCAGCMGCCGCGGTAA	926R: CCGYCAATTYMTTTRAGTTT	– 98°C, 30s – 30 denaturation cycles (98°C, 10 s), primer hybridization (52°C, 30 s), primer extension (72°C, 30 s) – Final extension step 72°C, 5 min	Parada et al. (2016)
Illumina MiSeq & NovaSeq	ITS2	MiSeq: 350 NovaSeq: 318	Fungi	Nested PCR PCR 1 ITS1: CTTGGTCATTTAGAGGAAGTAA	Nested PCR PCR1‐ITS4: TCCTCCGCTTATTGATATGC	– Same as V4–V5 16S rRNA but primer hybridization (57°C, 30 s)	Rämä et al. (2016)
				PCR 2 5,8S‐FUN: AACTTTYRRCAAYGGATCWCT	PCR 2 ITS4‐FUN: AGCCTCCCGCTTATGATATGCTTAART	– Same as V4–V5 16S rRNA but primer hybridization (58°C, 30 s)	Taylor et al. (2016)
ONT Mk1C	Full 16S rRNA	1413	Bacteria	27F: AGAGTTTGATCMTGGCTCAG	1492R: CGGTTACCTTGTTACGACTT	– Same as V4–V5 16S rRNA but primer hybridization (71°C, 30 s)	van der Loos et al. (2021)
	Full ITS	1326	Fungi	ITS9‐mum: TGTACACACCGCCCGTCG	LR3‐I: TGGTCCGTGTTTCAAGAC	– Same as V4–V5 16S rRNA but primer hybridization (62°C, 30 s)	Toju et al. (2012), Mafune et al. (2020)

Adaptive sampling enables the enrichment or depletion of specific reads in real‐time during sequencing. As it passes through the nanopore, the single DNA strand generates a measurable current, called a “squiggle,” which is compared to a reference sequence database. Depending on whether the signal matches a reference, the channel can temporarily reverse the voltage across its pore, thereby ejecting the undesired read and making the pore available for other molecules. Adaptive sampling using the “Readfish” approach has already been tested in metagenomics datasets: Martin et al. (2022), for instance, created a synthetic mock community of seven bacterial species at different abundances and demonstrated enrichment of low‐abundance species but also successfully depleted the bacterium *Morganella morganii* .

In the absence of comparative studies on seaweed samples, and in view of the challenges posed by the amplification of plastid reads inherent to this type of sample, it is essential to compare different approaches for characterising the microbiota associated with seaweeds. Here, we explore the bacterial and fungal communities associated with the brown macroalga *A. nodosum*. We study 28 samples through different metabarcoding approaches to (i) evaluate the efficiency of the “Readfish” approach to remove plastid reads, (ii) determine which technology (MiSeq, NovaSeq, and Mk1C sequencers) provides the best measure of microbial diversity associated with *A. nodosum* while also assessing how the choice of assignment methods and primers affects the results, particularly considering the issue of plastid reads, and (iii) highlight the advantages of combining Illumina and ONT reads to taxonomically assign unknown fungi.

## 2 Materials and Methods

### 2.1 Sample Collection

**FIGURE 1:**
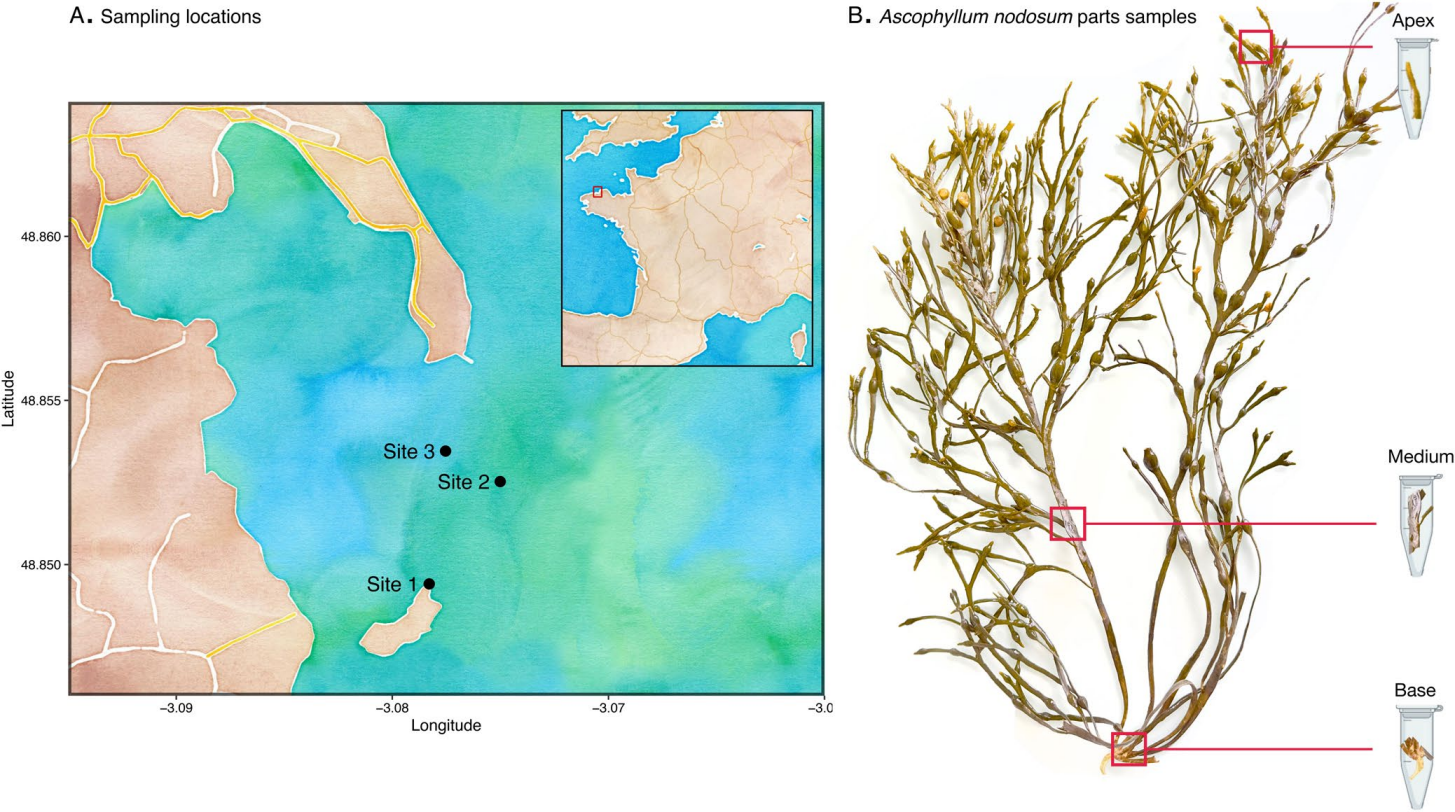
Sampling locations. (A) The maps show the location of the selected sites at Pleubian, Brittany (Site 1: 48.849411, −3.078291, Site 2: 48.852532, −3.075012, Site 3: 48.853465, −3.077533). The three samples are close to each other (Site 1‐Site 3: 354 m, Site 1‐Site 2: 386 m, Site 3‐Site 2:180 m. (B) The figure illustrates the three parts of the *Ascophyllum nodosum* sampled (apical, medium, and basal).

To compare the three sequencing methods, the sampling was carried out at Pleubian (Brittany, France) on 2 November 2021, and 22 March 2022, in three nearby sampling sites (Figure 1A). At each of these sites, four algae were chosen randomly. For each individual, three parts were sampled: apex, medium, and basal parts (Figure 1B). Small algal samples (approximately 50 mm long) were cut with clean scissors rinsed with 70% ethanol, immediately placed in cryotubes, and kept on ice during sampling. Once at the laboratory, samples were stored at −80°C.

### 2.2 Microbial DNA Extraction of *A. nodosum*

To extract microbial DNA from *A. nodosum* , algal samples were freeze‐dried (Cryo Rivoire, Montpellier) for 48 h and 10 mg of dry sample was then pre‐ground using a mortar and more finely ground with a TissueLyser II bead beater (1 min, 30 Hz) (Qiagen, Hilden, Germany). Polyphenolic compounds were removed twice by washing the samples with 1 mL of acetone 100%. The mixture was vortexed for 10 min followed by 1 min of centrifugation at 12,800 g (room temperature), and the liquid phase was removed. The bead needed for the TissueLyser II was removed, and nucleic acids were extracted using 1 mL of 2% CTAB extraction buffer (2% CTAB, 2% PVPP, 1.4 M NaCl, 20 mM EDTA [pH 8], and 100 mM Tris–HCl [pH 8]). Then, 35 μL of DIECA 0.1 M (ref. 228680, Sigma‐Aldrich) and 10 μL of β‐mercaptoethanol (ref. M31148, Sigma‐Aldrich) were added, and samples were incubated for 1 h at 65°C (Panova et al. 2016). Then, 1 mL of chloroform/isoamyl (24:1) was added (10 min shaker, 30 min of centrifugation at 18,506 g) (Varela‐Alvarez et al. 2006), and 750 μL of the upper (aqueous) phase was recovered. Polysaccharides were precipitated by adding 225 μL of 100% ethanol drop by drop. Then, 975 μL of chloroform was added (Bernard et al. 2018). After 1 min of shaking and 20 min of centrifugation at 18,506 g, 550 μL of supernatant was collected. DNA was precipitated with 110 μL of 3 M sodium acetate and 1.1 mL of pure ethanol for 1 h at −80°C, followed by 30 min of centrifugation at 18,506 g (4°C). The supernatant was removed, and the DNA pellet was washed twice with 500 μL of 70% cold ethanol (20 s shaker, 10 min of centrifugation at 18,506 g, 4°C). The DNA pellet was dried at room temperature and resuspended in 450 μL of molecular biology‐grade water (ref. 46‐000‐CV, Corning, USA). DNA was then further purified with a NucleoSpin Plant II kit (MachereyNagel, Germany) according to the manufacturer's instructions, except that cleaning with the PW2 buffer was carried out twice. Finally, a third purification was performed using AMPure XP Beads (Beckman Coulter, Brea, CA, USA). DNA quality was estimated with the Nanodrop (Nanodrop One, ThermoFisher) and DNA concentration with the Qubit dsDNA BR Assay Kits (Thermo Fisher, USA). The eluted DNA was stored at −20°C. This DNA extraction is available in protocols.io (dx.doi.org/10.17504/protocols.io.8epv52kx6v1b/v1).

### 2.3 DNA Amplifications

The DNA extracted from the three sampled parts (apex, medium, and base) of an individual thallus was pooled at equal concentrations, and the 28 pools were diluted to 1 ng/μL final concentration. Two equal bacterial and fungal mock communities containing DNAs from 10 bacterial species (*Paraglaciecola mesophila*, *Cobetia litoralis*, *Vibrio splendidus* , *Sulfitobacter undariae*, *Pseudoalteromonas marina* , *Roseovarius nanhaiticus* , *Marinomonas ushuaiensis* , *Ruegeria meonggei*, *Leucothrix pacifica*, *Bacillus safensis* ) and from 10 fungal species at equal concentrations (*Penicillium* sp. AN122R, *Penicillium* sp. ANF312, *Hypocreales* sp. LDF120R, *Hypocreales* sp. ANF130H, *Hypocreales* sp. LD40H, *Botryotinia fuckeliana* SLF474T, *Dendryphion penicillatum* ANF44R, *Microsphaeropsis olivacea* LDF50, *Trametes versicolor* ANF131, *Phaeosphaeria* sp. ANF596H) were generated and diluted to 1 ng/μL final concentration. Two negative controls containing all reagents used during DNA extractions were added.

**FIGURE 2:**
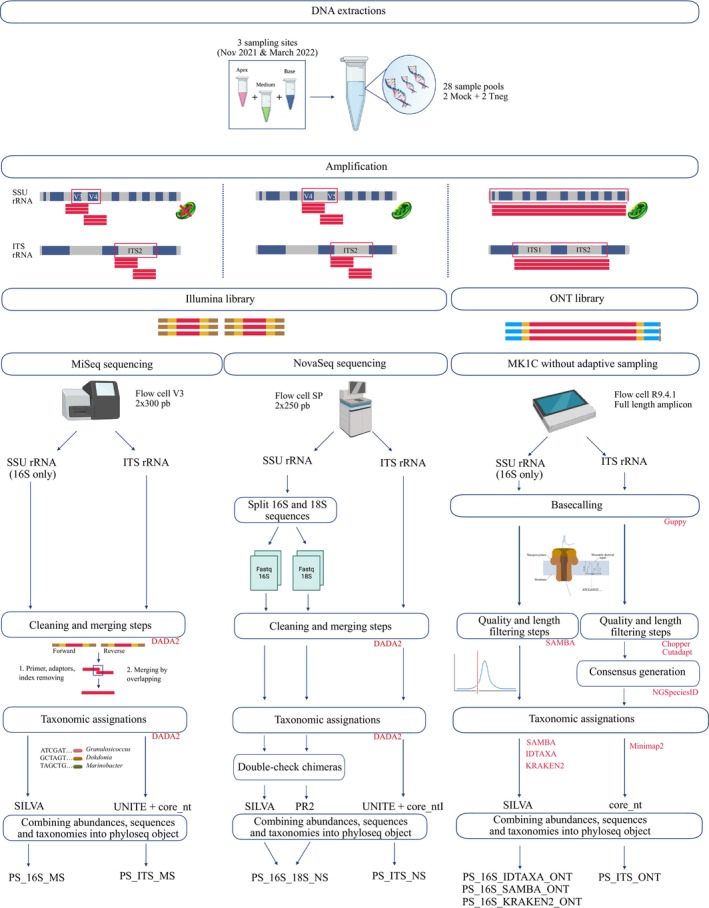
Primers and the bioinformatic processes used for each sequencing technology. The MiSeq primers are the only 16S primers that do not amplify plastid DNA. Red colour indicates the tools used. The names used in the last row illustrate the phyloseq objects were generated during the analysis. They are generic and not used elsewhere in the manuscript. (“MS” = MiSeq, “NS” = NovaSeq, “PS” = Phyloseq object).

To comprehensively describe the microbiome of *A. nodosum* , we selected different sequencers (MiSeq, NovaSeq, Mk1C—i.e., ONT) and primer pairs. The primers used for MiSeq and ONT sequencing targeted the bacterial communities, while the universal primers used for NovaSeq sequencing targeted bacterial, archaeal, and eukaryotic communities in the same sequencing run (Table 1). For all sequencing types, PCRs were performed in duplicate in 12.5 μL reaction mixtures containing 6.25 μL of Q5 High‐Fidelity 2× Master Mix (New England BioLabs, MA, USA), 0.25 μL of each primer at 10 nM, 1.25 μL TBT‐PAR 10× mix (Samarakoon et al. 2013) and 3.1 μL of diluted DNA template at 1 ng/μL. For fungal amplification, a second PCR was carried out using 3.1 μL of the first PCR product diluted to 1:100. The PCR cycling conditions are reported in Table 1. PCR products were assessed by gel electrophoresis, pooled, and stored at −20°C (Figure 2).

### 2.4 Library Preparations and Sequencing

#### 2.4.1 Illumina

Each PCR product (12.5 μL) was purified with 10 μL of NucleoMag magnetic beads (Macherey‐Nagel) according to the manufacturer's recommendations. Amplicons were eluted in 27.5 μL of 10 mM Tris–HCl (pH 8.5). Amplicons were indexed using the Nextera XT Index V3 set D kit (ref. 20531058, Illumina, San Diego, CA, USA) for the MiSeq sequencing and with the IDT for Illumina DNA/RNA UD indexes Set A, B, C, and D Tagmentation (96 indexes, 96 samples) (refs. 20027213, 20027214, 20042666 and 20042667) for NovaSeq sequencing. Library amplifications were performed in 50 μL reaction volumes containing 25 μL of Q5 High‐Fidelity 2× Master Mix (New England BioLabs, MA, USA), 5 μL of each Nextera XT index for MiSeq or 10 μL of IDT for Illumina DNA/RNA UD indexes for NovaSeq, 10 μL of water, and 5 μL of the amplicon. After an automated NucleoMag cleanup using an ML‐Star robot (Hamilton), the mean fragment size was estimated with a LabChip GX Touch (Perkin Elmer, MA, USA), and libraries were quantified with the Quantifluor ds DNA system (Promega, WI, USA). MiSeq sequencing was performed at the Genomer platform (Station Biologique de Roscoff, France) using a MiSeq Reagent Kit v3 (2 × 300 bp) (ref. MS‐102‐3003). NovaSeq sequencing was performed at the Genomics Core Facility GenoA platform (Nantes, France) using a NovaSeq 6000 SP Reagent Kit 500 cycles v.1.5 (ref. 20028402).

#### 2.4.2 Oxford Nanopore Technologies

Each PCR product (15 μL) was purified with 12.5 μL AMPure XP Bead (Beckman Coulter, Brea, CA, USA) according to the manufacturer's recommendations. DNA was quantified with the Qubit 1× dsDNA High Sensitivity Assay kit (Thermo Fisher, USA). Indexing was performed by using 16 μL of purified DNA amplicon diluted in 8 μL of molecular biology‐grade water (ref. 46‐000‐CV, Corning, USA). A PCR mix was prepared with 1 μL of barcodes (PCR Barcoding Expansion 1–96, EXP‐PBC096, Nanopore), 24 μL of diluted DNA amplicon, and 25 μL of Q5 High‐Fidelity 2× Master Mix (New England BioLabs, MA, USA). The PCR cycling conditions were 95°C for 3 min followed by 15 cycles of denaturation (95°C, 15 s), primer hybridisation (62°C, 15 s), primer extension (65°C, 45 s), and a final extension step of 65°C for 2 min. Then, PCR products were purified with 22.5 μL of AMPure XP Beads (ratio 0.45) and quantified with the Qubit dsDNA High Sensitivity Assay Kit (Thermo Fisher, USA). The mean fragment size was estimated using a LabChip GX Touch (Perkin Elmer, MA, USA), and all samples were normalised to 17 ng/μL and pooled in an Eppendorf DNA LoBind Tube. A 47‐μL aliquot of this pool was used for the ONT sequencing without adaptive sampling and another one for ONT sequencing with adaptive sampling. DNA repair, DNA purification, DNA ligation, and DNA quantification were carried out according to the standard Nanopore protocol (Ligation sequencing amplicons—PCR barcoding: SQK‐LSK109 with EXP‐PBC096). The R9.4.1 SpotON flow cell was loaded according to the Nanopore recommendations, and sequencing was performed using a MinION Mk1C (Oxford Nanopore) for 72 h. The plastid sequence (contigs A‐nodosum_M_contig2489_93747_95239 and A‐nodosum_M_contig2489_18795_17302) found in *A. nodosum* genome from the Phaeoexplorer project (https://phaeoexplorer.sb‐roscoff.fr/organism/ascophyllum‐nodosum_male/) was uploaded to the Mk1C sequencer for the adaptive sampling run.

### 2.5 Bioinformatic Analysis

The bioinformatic analyses were tailored to the different sequencing platforms and their particularities, in particular the availability of eukaryote sequences for the NovaSeq and the high error rate in the ONT data. Our strategy was to analyse each dataset using the optimal pipeline rather than finding a single pipeline, and thus basing our comparisons on the best possible results.

#### 2.5.1 MiSeq

Sequence quality was visually inspected with FastQC v.11.9 (https://www.bioinformatics.babraham.ac.uk/projects/fastqc/), and forward and reverse primers were trimmed for the ITS (i.e., fungal sequences) with Cutadapt v.4.0 (Martin 2011) and 16S (i.e., bacterial sequences) using the trimLeft option of the FilterAndTrim function from the DADA2 package. Each set of sequences was filtered (FilterAndTrim function—16S rDNA: maxN = 0, maxEE = c(2, 2), truncLen = c(300, 200), truncQ = 2, trimLeft = c(17, 19) and ITS: maxN = 0, maxEE = c(2, 5), truncLen = c(260, 230), truncQ = 2, minLen = 50), merged, and combined into Amplicon Sequence Variants (ASVs) using the DADA2 package v1.26.0 (Callahan et al. 2016) in R v. 4.2.3. The 16S and ITS ASVs were taxonomically assigned with a naive Bayesian classifier method (“assignTaxonomy” function from the dada2 package) using the non‐redundant small subunit database v.138.1 (Quast et al. 2013) and the UNITE database v.9 (release 07.25.2023, Kõljalg et al. 2013), respectively. To complement this taxonomic assignment, unclassified sequences were manually compared to the online NCBI core_nt database with Blastn (https://blast.ncbi.nlm.nih.gov/), between November 2023 and August 2024, and the taxonomy of the best target sequence was assigned to the ASV if the sequence identity was at least 98% with an Evalue ≤ 1e−50 and a query coverage > 95%. These classifications are marked with the suffix “Blast” in this article. For both 16S and ITS, ASVs, taxonomic assignments, and contextual data were combined into a phyloseq object using the Phyloseq package v1.44.0 (McMurdie and Holmes 2013) and decontaminated using extraction controls with the microDecon package with the *thresh* = 1 option (v1.0.2) (McKnight et al. 2019).

#### 2.5.2 NovaSeq

Sequences were quality‐controlled, and primers were removed with Cutadapt. Sequence analysis differed from that used for the MiSeq sequencer (which only targets the 16S rRNA gene), as we used universal primers that amplify both prokaryotes and eukaryotes from the SSU rRNA gene (Parada et al. 2016). The analysis was carried out following the 16S and 18S (i.e., eukaryotic) mixed online pipeline (https://astrobiomike.github.io/amplicon/16S_and_18S_mixed). To separate the 16S and 18S, the SSU fastq files were aligned to the 18S PR2 database using the magicblast tool v.1.5.0. Each set of 16S and 18S fastq files was filtered independently (16S + 18S—FilterAndTrim function: maxN = 0, maxEE = c(2, 2), truncLen = c(231, 230), truncQ = 2 and ITS—maxN = 0, maxEE = c(2, 2), truncQ = 2), merged for 16S, concatenated for 18S, and denoised into ASVs with the DADA2 package v1.26.0 (Callahan et al. 2016) in R v.4.2.3. 16S, 18S, and ITS ASVs were taxonomically assigned using the non‐redundant SILVA small subunit database v.138.1, the PR2 database v.5.0.0 (Guillou et al. 2013), and the UNITE database v.9, respectively. Taxonomy was also completed with Blastn searches against the NCBI database, as in the MiSeq analysis. Bacterial (16S), eukaryotic (18S), and fungal (ITS) ASVs were combined in phyloseq objects (v1.44.0). Bacteria and eukaryote ASVs shorter than 350 bp in length were removed, and 16S ASV tables were decontaminated using extraction controls with the microDecon package (thresh = 1) (v1.0.2), whereas ITS ASVs were not decontaminated due to problems with the negative control samples. All sequences containing homopolymers longer than five bases were removed. We also detected chimaeras in low abundances, which distorted the eukaryotic composition. To remove the chimaeras that remained despite the DADA2 chimaera detection, we performed a second identification of chimaeras using vsearch v.2.22.1 with the*—uchime_ref* option and the SILVA non‐redundant small subunit database v.138.1 as reference.

#### 2.5.3 Oxford Nanopore Technologies

For ONT sequencing, two independent analyses were carried out. A quick analysis to evaluate the adaptive sampling efficiency and an in‐depth analysis with the most promising ONT run to provide maximum comparability with the analyses carried out for the Illumina data.

For the quick method, we performed the analyses according to the default parameters implemented in the software of the Mk1C sequencer v.22.05.8 (real‐time basecalling with high accuracy Guppy v.6.1.5, https://github.com/nanoporetech/rerio) (Wick et al. 2019). The SAMBA workflow v.4 (https://github.com/ifremer‐bioinformatics/samba) for long‐read metabarcoding was used on the raw reads to be in the same condition as for real‐time read ejection, and the taxonomic assignment in the SAMBA's workflow was performed with the non‐redundant SILVA small subunit database v.138.1 (Quast et al. 2013).

The in‐depth method was only applied to the run without adaptive sampling and was used to compare the sequencing methods based on bacterial (16S rRNA gene) and fungal (ITS rRNA gene) amplification. Super accuracy basecalling was redone using Guppy v.6.4.6, and sequencing quality was estimated with NanoPlot v.1.42.0 (De Coster et al. 2018). For read processing, we compared different strategies for the bacterial dataset, while we only tested consensus generations for the fungal dataset because many sequences were unassigned despite searches in the NCBI core nt database. Obtaining a high‐quality full‐length ITS rRNA gene enabled us to target diverse references from all rRNA regions available in public databases.

For bacterial read processing, primers and reads with an average quality score 13 were removed using Cutadapt. Only reads between 1000 and 2000 bp were further analysed using the SAMBA pipeline v.4 (https://gitlab.ifremer.fr/bioinfo/workflows/samba). A comparison of taxonomic assignment methods was performed between the IDTAXA function from the DECIPHER R package, which is a k‐mer‐based classifier (threshold = 50, MinDescend = 0.9) (Murali et al. 2018), the SAMBA workflow, where custom taxonomic assignment is performed by sequence mapping against the taxonomic database (minimap2 tool), and KRAKEN2 v.2.1.2, which also uses k‐mers (Wood et al. 2019) with the non‐redundant SILVA small subunit database v.138.1. The average number of reads per sample was approximately 85,000 for bacterial ONT sequencing, with a maximum and a minimum of 256,408 and 43 reads, respectively. We confirmed that these two extreme samples in terms of the number of reads were outliers using the Grubbs test in Past4 v.4.15 (Hammer et al. 2001), and we suspect that this may have been caused by a pipetting error during the indexing step. Both samples were therefore removed for the rest of the analyses.

The generation of consensus sequences (i.e., representative sequences derived from the alignment of multiple related sequences) was a great option to decrease Nanopore sequencing errors. First, 16S and ITS sequences with a length between 1000 and 2000 bp and a *Q*‐score greater than 10 were kept with Chopper v.0.7.0 (De Coster and Rademakers 2023). We chose a lower *Q*‐score than above because sequences will be corrected with the consensus method. Primers and sequences without primers were removed using Cutadapt v.4.5(*—e = 0.2* and*—revcomp* options). ONT sequencing errors were polished by producing consensus sequences with the NGspeciesID pipeline v.0.3.0 (Sahlin et al. 2021). The pipeline was run after concatenating each cleaned sample file with an abundance ratio threshold of 0.002. The consensus was polished with Racon using six iterations. To obtain an abundance table based on read counts sample, we aligned all reads from each sample against the consensus sequences with minimap2 and the *‐x ava‐ont option* v.2.24 (Li 2018). The read counts were then obtained using samtools *idstats* v.1.18 (https://www.htslib.org/). All reads belonging to *A. nodosum* were removed.

Consensus sequences were aligned against mock community references as a positive control using the mafft online server v.7 (https://mafft.cbrc.jp/alignment/server/index.html) with the G‐INS‐I iterative strategy. The aligned sequences were curated with Gblocks, and phylogenetic trees were estimated using PhyML (SH‐LIKE, GTR substitution model) on the phylogeny.fr server (Dereeper et al. 2008).

MiSeq, NovaSeq, and ONT phyloseq objects were merged and normalised by the median. Unassigned sequences in Illumina sequencing were identified by aligning short reads and consensus sequences from ONT. Barplots were generated with ggplot2 v.3.5.0 (Wickham 2016). Sequencing strategy and bioinformatic pipelines are summarised in Figure 2. The similarity between the three sequencing runs was estimated with the Renkonen similarity index on class‐level classifications (Goldford et al. 2018). The bioinformatic scripts used in this article are available via GitHub: https://github.com/rssco/Illumina_ONT_comparisons.

## 3 Results

### 3.1 Real‐Time Removal of Plastid Reads Truncates Plastid and Some Bacterial Reads Without Increasing Throughput for Other Bacteria

**FIGURE 3:**
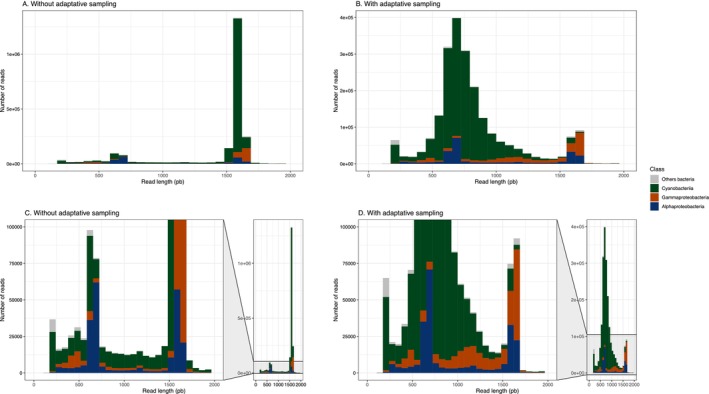
Comparison of sequence length distributions between the ONT sequences without adaptive sampling (A) and without adaptive sampling (B). The colours show the taxonomy at class level. Figures (C) and (D) represent a zoom of the number of reads between 0 and 10,000 reads.

The ONT (long‐read sequencing) produced a similar number of reads both without (2.52 M reads) and with adaptive sampling (2.49 M reads). The mean length was lower with the adaptive sampling method (756 bp) than without (1291 bp). The number of reads taxonomically identified as plastid reads was 1,731,689 (69%) without adaptive sampling and 1,700,150 (68%) with adaptive sampling. In the dataset obtained without adaptive sampling, the majority of plastid sequences identified in the class Cyanobacteria were around 1500 bp (Figure 3A). On the other hand, in ONT sequencing with adaptive sampling, they had different sizes (mostly between 700 and 800 bp, Figure 3B). Another observation was the different read size distributions for Alphaproteobacteria and Gammaproteobacteria with the adaptive sampling method (Figure 3C,D). There were more reads between 900 and 1000 bp for the Gammaproteobacteria and more small reads around 700–800 bp for the Proteobacteria compared to the dataset without adaptive sampling. Given these results, all subsequent comparisons were conducted using the ONT run without adaptive sampling.

### 3.2 Wide Variability in the Number of Final Reads After the Cleaning and Filtering Steps

**FIGURE 4:**
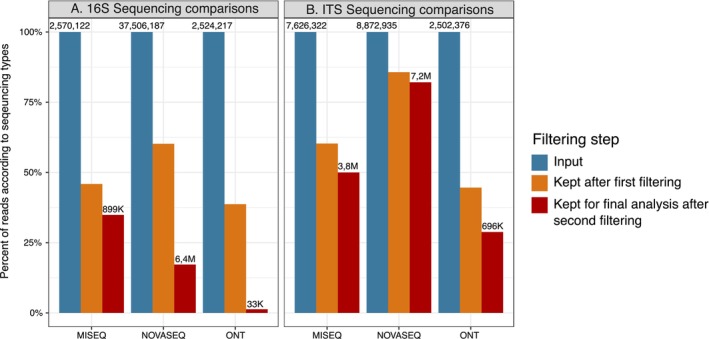
Percent of reads kept during the analyses of (A) 16S and (B) ITS sequencing comparisons where ONT represents the SAMBA and NGSpeciesID pipelines, the different classification methods used. Blue indicates the raw reads (always 100%), orange the proportion of reads kept after a first round of read filtering (length and quality filtering, and the DADA2 processes if used), and red indicates the proportion of reads after second filtering step (decontamination, host, plastid, chimaera, and homopolymer removal).

Regarding the SSU (i.e., bacteria, archaea, eukaryotes) amplicon sequencing results for our 28 seaweed samples, 2 mock communities, and 2 negative controls, we obtained the highest number of read pairs with the NovaSeq sequencing (about 37 M including 18S reads, i.e., Illumina, short‐read sequencing), whereas the MiSeq (i.e., Illumina, short‐read sequencing) produced about 2.5 M read pairs, and the Mk1C sequencing (i.e., ONT, long‐read sequencing) generated about 2.5 M reads (Figure 4A). The mean number of reads (± SD) per sample was 1,339,507 (± 423,827); 91,790 (± 25,477); and 90,151 (± 42,717) for NovaSeq, MiSeq, and ONT sequencing, respectively. However, both Illumina sequencing runs were shared with other projects, so that the read counts reported here do not reflect the full capacity of an Illumina run. The first step of filtering eliminated many reads in the ONT run: only 39% were retained because the majority of reads had a *Q*‐score < 13 (54% of the total reads). MiSeq sequencing yields better read quality, especially for forward reads, resulting in almost 50% of the total reads being retained after the first quality filter. Reads were mostly lost during quality trimming and chimaera detection for both Illumina runs. The MiSeq sequencing yielded the highest percentage of target reads after the second round of filtering (decontamination, host‐, plastid‐, chimaera‐, and homopolymer removal) because no plastid reads were amplified by the primer, followed by the NovaSeq run with 12%. Almost all reads were removed after the suppression of plastid reads for the ONT runs, leaving only 1.2% and 2% of the total reads.

In general, a higher percentage of ITS (i.e., fungal) reads were retained because there were no plastid reads to remove (Figure 4B). The mean number of reads (± SD) per sample was 316,891 (± 146,641); 272,369 (± 61,570); and 89,371 (± 26,755) for NovaSeq; MiSeq; and ONT sequencing, respectively. After the first read filtering, 60%, 86%, and 45% of the total reads were retained for MiSeq, NovaSeq, and ONT sequencing, respectively. Then, 82% of the total reads remained after the second filtering for the NovaSeq, 50% for the MiSeq, and 29% for ONT. In the ONT run, half of the reads were between 1000 and 2000 bp with a *Q*‐score > 10.

### 3.3 A Comparison of Bacterial Assignment Methods for ONT Shows Biases According to the Analysis Pipeline

**FIGURE 5:**
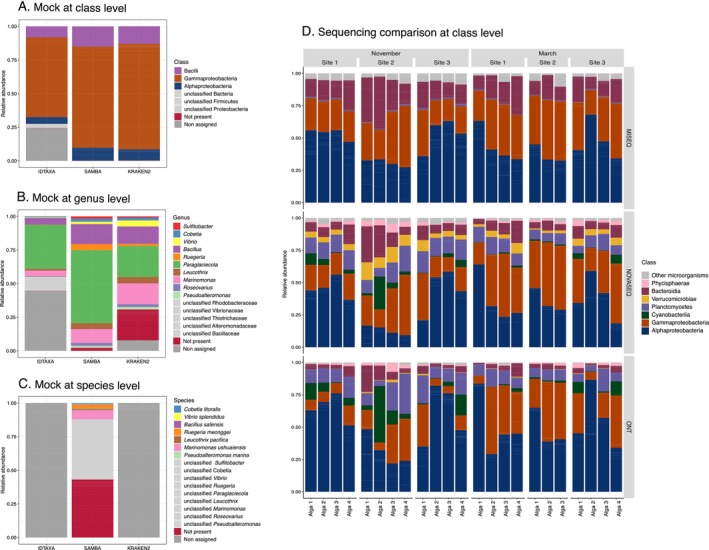
Panels (A–C) show analyses of the bacterial mock communities in relative abundance through different methods of nanopore assignments. The assignments with IDTAXA, KRAKEN2, and SAMBA were compared at class (A), genus (B), and species (C) levels. The red colour corresponds to assigned sequences that were not part of the mock community. Panel (D) shows a comparison based on the *A. nodosum* field samples at class level.

We compared four pipelines (IDTAXA, KRAKEN2, SAMBA, and NGSpecies) to determine the most accurate classification pipeline for bacterial ONT sequences using the bacterial (i.e., 16S) mock community. While the NGSpecies pipeline generated consensus sequences that could minimise sequencing errors, these consensuses diverged from the actual bacterial sequences present in the mock community, with a maximum identity of only 97.5% (Figure S1). This approach was not suitable for accurately classifying 16S sequences. Concerning the KRAKEN2 and SAMBA pipelines, the classification at the class level was highly similar with a high abundance of Gammaproteobacteria (Figure 5A). The IDTAXA pipeline exhibited the highest percentage of unassigned reads compared to the two other methods. At lower taxonomic ranks, there were differences in the detected mock community between all three assignment methods. The 10 bacterial genera were well assigned with SAMBA and KRAKEN2, with a higher percentage of *Paraglaciecola* and *Ruegeria* in the SAMBA assignment. IDTAXA was not able to assign *Sulfitobacter* and *Pseudoalteromonas* and again presented a higher percentage of unassigned sequences at the genus level, but also a lower percentage of false positive classifications (i.e., genera not present in the sample) (Figure 5B). SAMBA was able to assign seven bacteria at the species level but with a large proportion of false positive assignments (Figure 5C). IDTAXA and KRAKEN2 were not trained to make species level assignments. Even if a few false positives were detected in the SAMBA classification method, SAMBA provided the highest taxonomic resolution at the genus level. Therefore, for the sequencing comparisons, the 16S ONT sequences were assigned using SAMBA.

### 3.4 The Observed SSU Community Depended in Part on the Primers Used and the Sequencing Methods

When comparing the microbial communities detected in the seaweed field samples with the three sequencing types (MiSeq, NovaSeq, and Mk1C), clear differences were observed. Overall differences and similarities between sequencing methods were estimated by performing a class‐level comparison with the Renkonen similarity index (1 = same, 0 = different). For SSU, MiSeq and ONT sequencing were more similar to each other (0.68, Figure S3A) than they were to the NovaSeq data (0.51 and 0.59). These differences are likely to be largely due to the different primer pairs used with the different sequencing methods. To assess this influence of the primer pairs, we performed an *in silico* PCR on the SILVA database NR SSU v.138.2, using the online tool SILVA TestPrime 1.0 (Klindworth et al. 2013). The universal primer pairs used for NovaSeq sequencing amplify both eukaryotes, bacteria, and archaea, whereas the primer pairs used for MiSeq sequencing targeted bacteria and archaea, and ONT targeted only bacteria (Table S1). Eukaryotes represented a small proportion with a dominance of the Ascomycota class (not counting reads from *Ascophyllum*) and were merged into “Other microorganisms” (Figure 5D). The primers used for MiSeq also did not amplify the Verrucomicrobia and Cyanobacteria classes well, with a predicted coverage of 54.8% and 5.6%, respectively (Table S2). Their absence is highlighted in MiSeq sequencing compared to other sequencing technologies (Figure 5D). In our mock community, we also observed the poor detection of the genera *Leucotrix*, *Pseuldoalteromonas*, and *Sulfibacter* in MiSeq sequencing (Figure S2). Verrucomicrobia was also not well detected in ONT compared to NovaSeq sequencing (83.9% vs. 94.2% predicted coverage, respectively).

Finally, we observed sequencing biases as planctomycetes were detected in very low abundance in MiSeq, where large proportions were observed in NovaSeq and ONT (Figure 5D). This observation likely does not stem from primer bias, as 91.3% of the Planctomycetes were expected to be detected with the primer used for MiSeq (Table S2).

### 3.5 The Combination of Both Illumina and Nanopore Sequencing Helps to Increase the Fungal Taxonomic Assignation Rate

The ITS2 short‐read sequencing (MiSeq and NovaSeq) showed a poor classification rate with the UNITE database, with approximately 64% of the unassigned ASVs at the class level, which represents 80% of the total reads in the MiSeq sequencing (Figure S4A). A part of these unknown sequences was assigned thanks to the NCBI databases, but many sequences remained unassigned because no corresponding ITS2 reference was available in public databases (“Uncultured,” Figure S4B).

**FIGURE 6:**
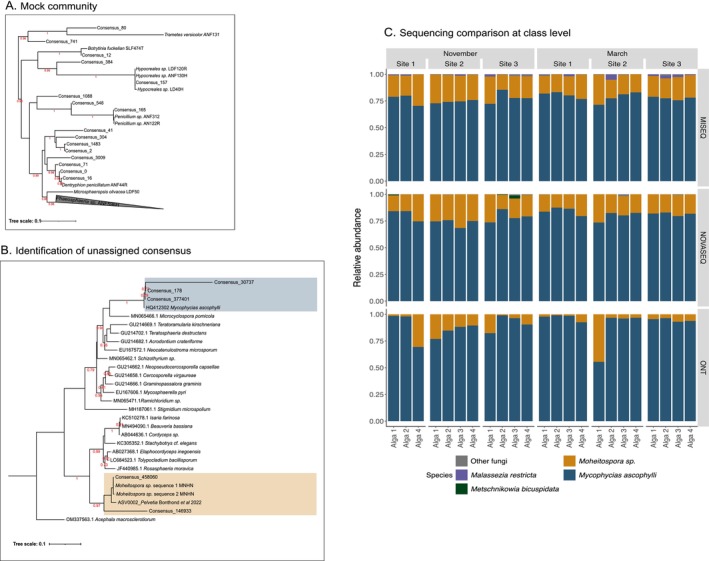
(A) Phylogenetic tree of ONT consensus sequences generated by the NGSpeciesID pipeline for the fungal mock community samples and the reference sequences. The grey triangle concerning *Phaeophaeria* sp. ANF596H corresponds to multiple ITS references. (B) Phylogenetic tree of consensus assignments found in *Ascophyllum* samples together with their closest sequences in NCBI. One sequence of *Mycophycias ascophylli*, two sequences of *Moheitospora* sp. found in the receptacle of *Ascophyllum nodosum* (*Moheitospora* sp. sequence 1 and 2 MNHN) and an ITS2 ASV identified as *Moheitospora* sp. (ASV00002_Pelvetia_Bonthond et al. 2022) in Bonthond et al. 2022 were also added. Alignments were performed with MAFFT tool (G‐INS‐I iterative strategy), the curation with Gblocks (94 positions remain for A, and 885 for B), and the tree was reconstructed using PhyML (SH‐LIKE, GTR substitution model). Support values < 0.70 have been removed. (C) Comparison of fungal communities detected with the different sequencing methods at the species level. Algae 3 and 5 at Site 2 in November were removed from the comparison because they were not sequenced with all three methods.

To target additional ITS regions in public databases and to improve taxonomic assignment for short‐read sequencing, the use of ONT sequences was a solution as the full ITS region was sequenced. Generating ONT consensuses helped to decrease the rate of sequencing errors. We first validated the consensus generator of the NGSpeciesID pipeline using the 10 fungal species included in the mock community. NGSpeciesID identified all fungi present in the mock except *Microsphaeropsis olivaceae* LDF50 and *Phaeophycaeries* sp. ANF596H (Figure 6A; Table S3).

All in all, NGSpeciesID gave satisfactory results for the fungal mock community, and we extended this analysis to the entire ONT dataset. In the latter dataset, only five consensus sequences were detected in the *A. nodosum* samples (Figure 6B). These were aligned against NCBI references to assign them (Figure 6B). Three consensuses were assigned as *Mycophycias ascophylli*: consensus 377,401 shared 99.77% of identity with the *Mycophycias ascophylli* reference HQ4123301, consensus 178 shared 99.18% of identity, and consensus 30,737 shared 100%, with 30% query coverage (i.e., 100% of the target sequence).

Using these longer consensus sequences, we were also able to assign shorter MiSeq and NovaSeq reads that lacked corresponding entries in public ITS databases (Figure 6C). ONT and Illumina did not have the same fungal distribution across samples, whereas there was high concordance between MiSeq and NovaSeq, which is supported by the high Renkonen similarity index (0.96, Figure S3B). ONT sequencing only detected *Mycophycias ascophylli* and *Moheitospora* sp., whereas MiSeq and NovaSeq analyses revealed additional low abundance fungal taxa.

## 4 Discussion

### 4.1 Ejecting Plastid Reads in Real‐Time Sequencing With Metabarcoding Data Is Not Efficient With Default Parameters

The study of seaweed‐associated bacteria through metabarcoding analysis has the consequence of amplifying a large proportion of plastid sequences of the photosynthetic host (Figures 3 and 4). To avoid the design of specific long‐read primers, adaptive sampling (i.e., ReadFish) has previously been proposed as an alternative approach to eject plastid reads in real‐time sequencing (van der Loos et al. 2021). Based on our experimental comparisons, we conclude that adaptive sampling with the Mk1C sequencer implemented with the MinKNOW software is not suitable for the removal of plastid sequences in 16S metabarcoding analyses. One problem is that the adaptive sequencing did not lead to an increase in the number of target reads. The reason for this is the prefix of ~700 bases for each read before deciding to eject. This represents almost half of the target region (Figure 4D) and is largely due to the time needed to make the decision—about 1.1 s of sequencing at 450 bases per second (Payne et al. 2021). The second problem is that the default alignment identity causes the ejection of a large proportion of bacterial reads due to the similarity between bacterial and plastid reads (Figure 4D). When considering only reads between 1000 and 2000 bp from adaptive sampling, we underestimate the bacterial diversity of *A. nodosum* across the three sampling sites. The MinKNOW software currently does not allow the configuration of the prefix length, alignment identity, or minimum alignment length for decision‐making, but several alternative adaptive sampling algorithms with adjustable parameters could lead to improvements of these results. UNCALLED, Sigmap, and SquiggleNet classify DNA sequences directly from electrical signals (Kovaka et al. 2021; Zhang et al. 2021; Bao et al. 2021). ReadBouncer combines fast CPU and GPU base calling to convert the current signal into a nucleotide sequence (Ulrich et al. 2022) and uses longer read prefixes for the decision‐making algorithm to prevent false rejection decisions (Ulrich et al. 2024). In the future, optimising the software and parameters specifically for our application combined with the use of the newer R10.4.1 flow cells with lower error rates and lower translocation speeds (240 bp per second; Ni et al. 2023) may render real‐time plastid sequence removal more beneficial. Other perspectives are, for instance, the development of long‐read primer pairs that do not amplify plastid reads, the selective removal of plastid DNA prior to amplification, or blocking the amplification of plastid DNA using mechanisms such as peptide nucleic acid clamps (Hussain et al. 2025). However, care must be taken to ensure that such strategies do not exclude certain bacterial groups, which may bias community composition and reduce taxonomic coverage.

### 4.2 SAMBA Provided the Best Taxonomic Resolution at the Genus Level but Challenges in ONT Read Processing Pipelines Remain

Illumina sequencing benefits from many well‐established pipelines for read processing (Callahan et al. 2016; Bolyen et al. 2019; Bernard et al. 2021; Noel et al. 2021) that have been developed by large, active communities for several years. The use of ONT for metabarcoding analyses, on the other hand, is relatively new, and fewer studies have already used the technology and attempted to identify the best pipelines for read processing (Marino 2020; Leidenfrost et al. 2020; van der Loos et al. 2022; Baloğlu et al. 2021). ONT provides its cloud‐based tool called EPI2ME, which is a user‐friendly end‐to‐end data analysis service. However, there are a few important limitations, such as restricted usage to ONT customers and the lack of possibilities to customise default parameters for taxonomy assignment (minimum accuracy of 77%). Moreover, in the study of the bacterial community of the green seaweed *Ulva* sp., the comparison of the EPI2ME tool with the KRAKEN2 pipeline showed many false positive classifications with EPI2ME and a lower bacterial diversity (van der Loos et al. 2021). KRAKEN2, which was originally developed for metagenomic analysis (Wood et al. 2019), is fast and easy to use. However, in our study, KRAKEN2 did not yield satisfactory results, with many false positives at the genus level in the mock community (Figure 5B). On the other extreme, the IDTAXA algorithm from the R package DECIPHER was developed to avoid misclassification errors (Murali et al. 2018). However, due to the high rate of sequencing errors in the ONT data, IDTAXA was unable to assign nearly 45% of the total reads in the mock community at the genus level. Despite the rare false positives detected, IDTAXA was therefore not ideal for working with ONT sequences. A third available tool is SAMBA, a standardised and automated metabarcoding workflow using Nextflow (Di Tommaso et al. 2017) developed by the Ifremer Bioinformatics Platform (SeBiMER) (Noel et al. 2021). This workflow offers an all‐in‐one analysis with integrity checking, construction of high‐quality ASV tables, and statistical analyses for short‐ and long‐read metabarcoding. The long‐read part of the workflow is dedicated to ONT, and in our mock community, SAMBA provided the best taxonomic resolution at the genus level. However, contrary to previous comparative studies suggesting that ONT metabarcoding analyses can discriminate different species within a genus (Shin et al. 2016; Matsuo et al. 2021; Huggins et al. 2024), we do not recommend working at the species level with bacterial 16S sequences, as approximately 45% of the reads were misclassified (Figure 5C).

The k‐mer‐based classifiers (i.e., IDTAXA and KRAKEN2), unlike the similarity‐based approach (i.e., SAMBA), fail to find enough k‐mers to make confident taxonomic assignments, likely due to the many errors in the ONT sequences. One simple solution to overcome this sequencing error problem is to use the new chemistry of the flowcell R10.4 combined with the Dorado basecaller (« Dorado » 2022), which offers faster processing and more accurate results (Sereika et al. 2022). Another possibility is to perform cleaning at the level of the raw signals. These electrical signals represent the DNA sequences and should be species‐specific. By aligning them without reference, it should be possible to group raw signals that belong to the same species and perform a signal correction as a consensus of electrical currents. Today, multiple innovative methods allow the alignment of raw signal data to a reference sequence, such as UNCALLED (Kovaka et al. 2021) or Squigualiser, an interactive alignment interface with single‐base resolution (Samarakoon et al. 2024). These methods should be developed to work without reference. Another promising, yet more costly and still less widespread alternative to the high error rate of ONT data is the use of PacBio HiFi sequencing, which generates long, high‐fidelity reads with an accuracy comparable to short‐read Illumina data (Hon et al. 2020).

### 4.3 Primer and Sequencer Choices Have an Impact on Microbial Diversity Analysis

Primer choice is known to affect the bacterial diversity observed by sequencing. This is due, in part, to the fact that different primers may target different regions of the 16S rRNA gene, which contains hypervariable regions with different evolutionary rates (Liu et al. 2008; Schloss 2010; Soergel et al. 2012). However, it may also be related to amplification biases depending on the primer pairs (Tables S1 and S2). The NOCHL primers of Thomas et al. (2020), for instance, were designed for MiSeq sequencing to reduce the amplification of plastid sequences of seaweed or plant hosts. In seaweeds, plastid reads could represent more than 85% of the total reads. Depending on the number of samples, this is problematic for MiSeq‐based barcoding, where the maximum number of output reads per run is 40 million, and it is even more dramatic for ONT sequencing. For the latter, the low number of output reads (in our case, 2.5 million per run), a high proportion of low‐quality reads and losses during filtering, as well as the necessity to work with consensus sequences for reliable assignments, exacerbate these limitations. In our study, after the removal of plastid sequences (Figure 3A), only 32,000 exploitable reads remained at the end of the pipeline across all 28 samples. This allowed us to focus only on the most abundant microbes. Controlling amplification of plastid reads by using the NOCHL primers results in a significant increase in the number of exploitable reads but also leads to an underestimation of 70%–90% of the Planctomycetes, Verrucomicrobia, and Cyanobacteria groups (Thomas et al. 2020), which is also what we observed in Figure 5D. NovaSeq sequencing can generate up to 1.6 billion paired‐end reads with the flow cell SP (Modi et al. 2021), and these reads are generally more accurate than reads from a MiSeq (Ravi et al. 2018; Modi et al. 2021). The loss of a large proportion of reads after filtration here does not have the same impact on the detection of rare microbes, enabling us to choose very generic primers that also amplified 18S sequences (Parada et al. 2016) while still leaving sufficient reads to exploit.

While comparative sequencing analyses between ONT and Illumina show no significant difference in most microbiome studies of non‐photosynthetic hosts (Shin et al. 2016; Winand et al. 2019; Heikema et al. 2020; Matsuo et al. 2021; Low et al. 2021; Egeter et al. 2022; Stevens et al. 2023; van der Reis et al. 2023), the presence of plastids in seaweeds poses an additional challenge, making it more difficult to establish a gold standard approach. In our case study, the NovaSeq sequencing coupled with the universal primers provided the most interesting results to exhaustively describe the bacterial partners of the brown macroalga *A. nodosum* , but ONT sequencing had some advantages when sequencing the ITS regions of marine fungi. The choice of a sequencing platform for microbiome analyses should thus depend on the target organisms (photosynthetic or not) and on the number and type of samples (ground tissues or swabbed). NovaSeq is particularly suitable for targeting multiple taxonomic domains and to work with large sample numbers of ground tissue samples. MiSeq is efficient for smaller sample numbers, with specific primers that exclude plastid reads or for swab samples. ONT sequencing offers quick turnaround, cost‐effective sequencing for low sample numbers, and full‐length (consensus) sequences for the most abundant microbial partners. It is also beneficial for sequencing fragments with highly variable lengths, where merging of forward and reverse reads may be challenging (e.g., eukaryotes).

### 4.4 Illumina and Nanopore: The Key to Going Further From Poorly Assigned Fungal Datasets

So far, the study of marine fungal communities associated with seaweeds is not yet commonplace (Rousseau et al. 2024), which leads to poor marine references in the UNITE database (Hassett et al. 2019). In our study, the Illumina sequencing (i.e., MiSeq and NovaSeq) targets the ITS2 region, resulting in many unassigned sequences (Figure S4), while ONT sequencing targeted the full ITS region with parts of the large and small subunit (LSU, SSU). Here, the consensus sequences (i.e., ONT sequences) generated by the NGSpeciesID pipeline proved to be extremely useful because they included other regions of the ITS, increasing our chances of finding matches in the NCBI database searches. Ultimately, it was these consensus sequences that allowed us to determine that the two major fungi present in the *A. nodosum* microbiome were *Moheitospora* sp. and *Mycophycias ascophylli*. It remains to be tested how well ONT would perform with more diverse fungal communities. This example clearly shows how Illumina and ONT data can be complementary—and we conclude that whenever possible, the combination of Illumina and ONT is the most appropriate method to characterise unknown alga‐associated microbial communities with confidence.

## 5 Conclusions

In this study, we showed that the removal of plastid reads in real‐time sequencing with the “ReadFish” adaptive sampling was ineffective when using the default parameters implemented in the MinKNOW software. Approximately 700 bases were sequenced before a read was ejected, representing about half of the length of typical 16S reads. More problematically, the high similarity between plastidial and bacterial sequences led to the ejection of many bacterial DNA fragments. We also showed that many false positive classifications could be generated depending on the taxonomic assignment method used in 16S metabarcoding analysis. Because of the rate of sequencing errors in R9.4.1 with ONT sequencing, we do not recommend conducting analyses at the species level, regardless of the assignment method used; however, for abundant microorganisms, the use of consensus sequences can help overcome this limitation. We highlight and discuss the advantages and disadvantages of each sequencing method, suggest some prospects for improvement, and demonstrate how, for our fungal analyses with a poor reference database, the combination of Illumina and ONT sequencing allowed us to go further than either technology alone.

## Author Contributions

C.R., C.L., and S.M.D. designed and conceptualised the research. C.R. collected samples, performed laboratory work, analysed the data, and wrote the manuscript. N.H. contributed a new analytical tool and reviewed the manuscript. S.R. performed cultures and DNA extractions of bacteria and fungi included in the mock communities. G.T. and E.L. performed sequencing preparations using newly designed protocols. C.L. and S.M.D. collected samples, supervised the research, and reviewed the manuscript. All authors have read and approved the manuscript.

## Conflicts of Interest

The authors declare no conflicts of interest.

## Supporting information


Appendix S1

Appendix S2

## Data Availability

The raw sequence reads and metadata are available in the European Nucleotide Archive (ENA) database under BioProject PRJEB80221 and sample accession numbers ERS21049679–ERS21049706. ASV tables with final abundances, taxonomies, and sequences are available in Appendix S1. Scripts are available on GitHub (https://github.com/rssco/Illumina_ONT_comparisons).
