# Supplementary material for: A Practical Comparison of Short‐ and Long‐Read Metabarcoding Sequencing: Challenges and Solutions for Plastid Read Removal and Microbial Community Exploration of Seaweed Samples
Source: Mol Ecol Resour. 2025 Jun 4;25(7):e14129. doi: 10.1111/1755-0998.14129 (PMC12415815; doi:10.1111/1755-0998.14129)
Supplement: Supplementary file 2 — Appendix S2 [file MEN-25-e14129-s002.zip › Supinfo.docx]

# Supplementary figures

Supplementary Figure 1 | Phylogenetic tree ONT consensus sequences generated by the NGSpeciesID pipeline for the bacterial mock community and the reference sequences. The red percentage corresponds to the percentage of identity (evalues=0). Sequences were aligned with the MAFFT tool (G-INS-I iterative strategy), the curation was carried out with Gblocks (1138 positions remain) and the tree reconstructed using PhyML (SH-LIKE, GTR substitution model). Support values under 0.70 have been removed.

**
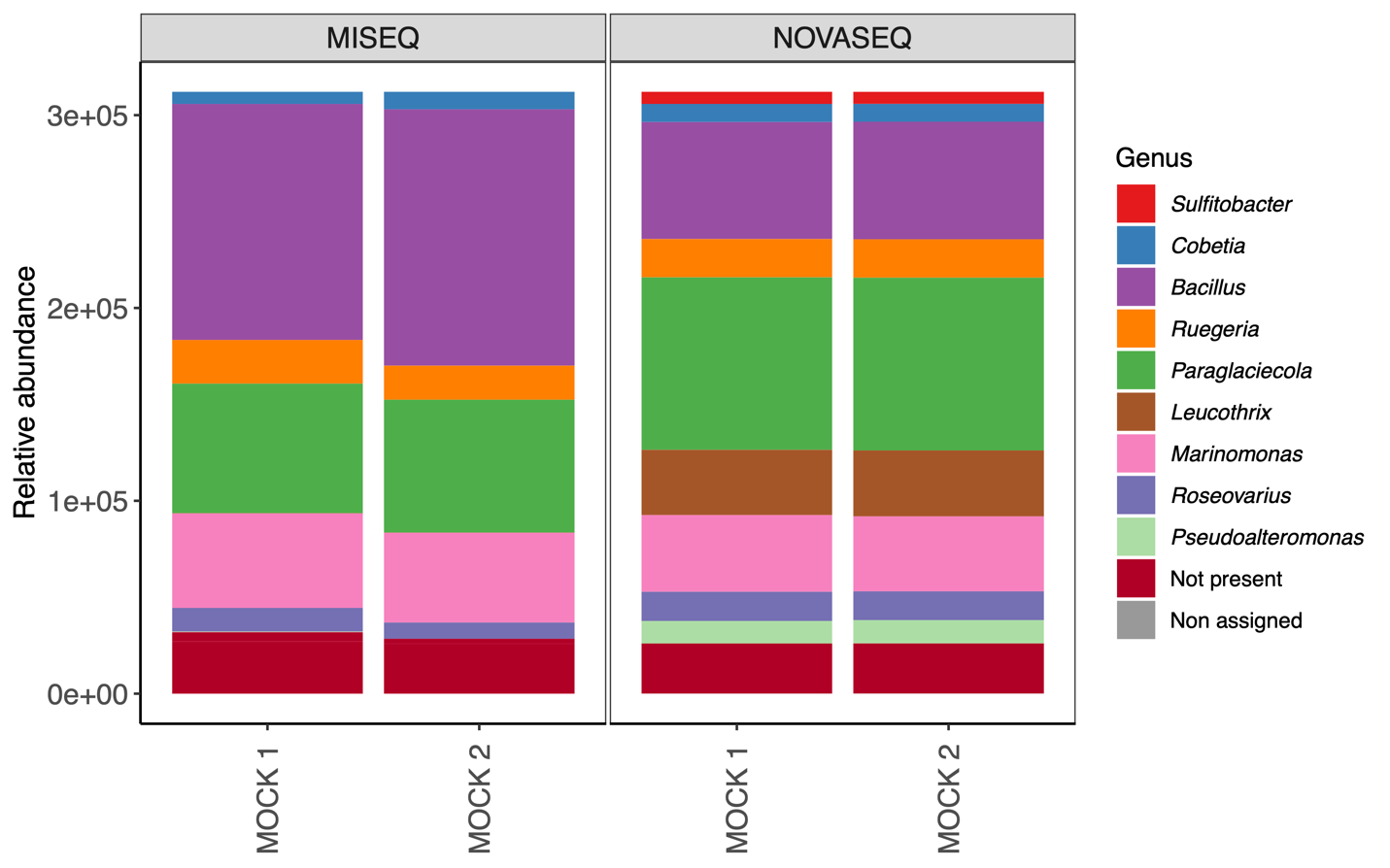
**

Supplementary Figure 2 | Analyses of bacterial mock communities through MiSeq and NovaSeq sequencing at genus level. The dark red color corresponds to assigned sequences that were not part of the mock community

Supplementary Figure 3 | Renkonen similarity index for SSU (A) and fungi (B) datasets at class-level comparison

**
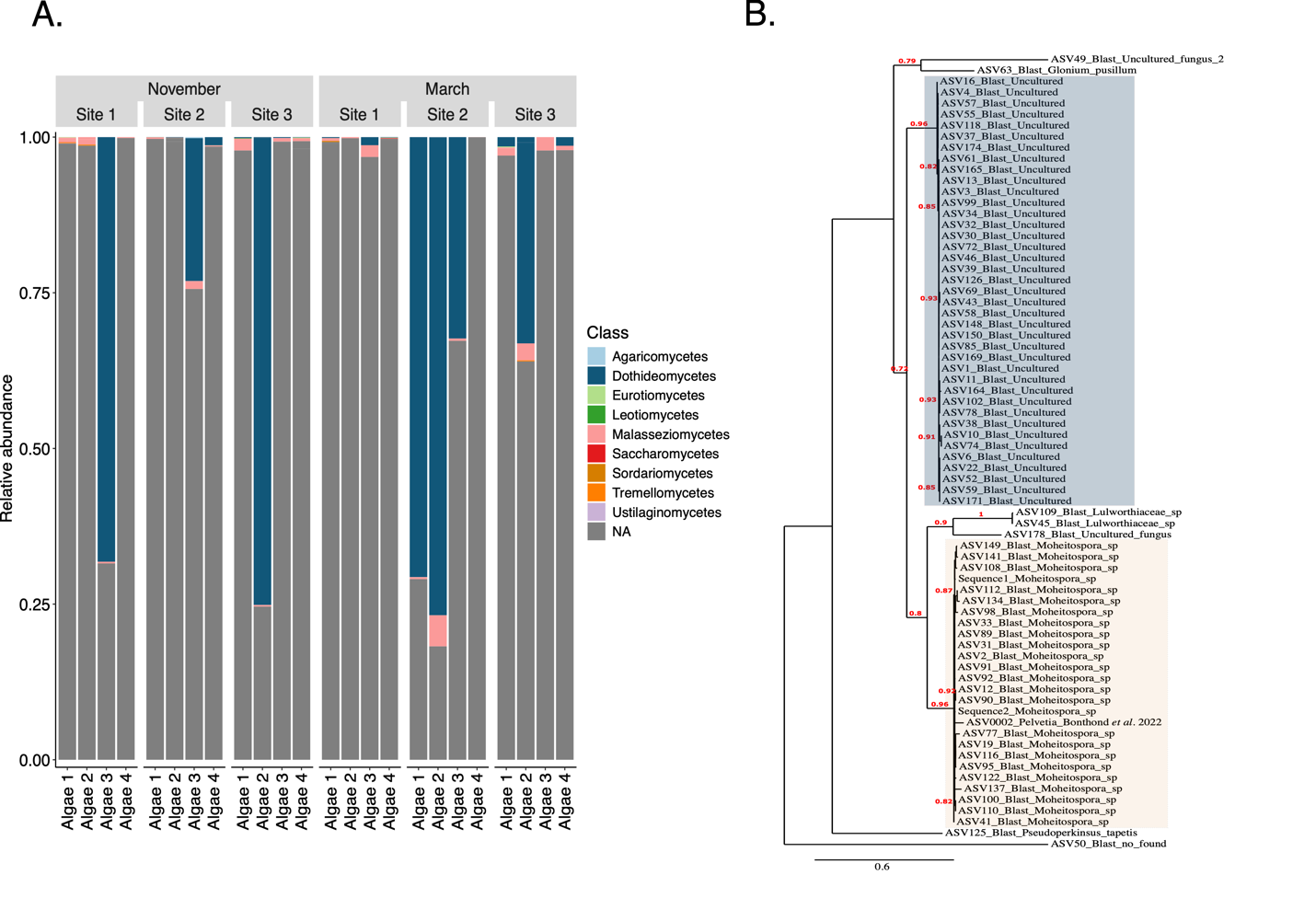
**

Supplementary Figure 4 | A) Bar plot showing all assigned and unassigned (NA) fungal classes in MiSeq sequencing. B) Phylogenetic tree of all unassigned MiSeq fungal sequences. The blue and yellow/beige shading correspond to the groups of “Blast_Uncultured” (e.g., unknown in NCBI) and *Moheitospora* sp. sequences, respectively*.* Two ITS2 sequences of *Moheitospora* sp*.* found in receptacle of *Ascophyllum nodosum* (Sequence1_ and Sequence2_Moheitospora_sp from MNHN) and one ITS2 ASV identified as *Moheitospora* sp*.* (ASV00002_Pelvetia_Bonthond *et al.* 2022) in Bonthond et al. 2022 were included as well. Alignment was performed with MAFFT (G-INS-I iterative strategy), curation with Gblocks (290 positions remained) and tree reconstruction with PhyML (SH-LIKE, GTR substitution model). Support values under 0.70 have been removed.

# Supplementary tables

Supplementary Table 1 | Predicted coverage of the primers used in this study at the kingdom level based on the sequences available in the SILVA SSU r138.2 database. Analyses were carried out using TestPrime 1.0 (2 mismatches per primer allowed with a no mismatch zone at the last two bases from 3’-end).

|  | NoCHL | Universal  Primer | Full-16S |
| --- | --- | --- | --- |
| Sequencing | MiSeq | NovaSeq | ONT |
| Bacteria | 89.7 | 93.1 | 80.2 |
| Archaea | 83.6 | 90.9 | 0.1 |
| Eukaryotes | 0.3 | 92.1 | 0.2 |

Supplementary Table 2 | Predicted coverage of the primers used in this study at the class level based on the sequences available in the SILVA SSU r138.2 database. Analyses were carried out using TestPrime 1.0 (2 mismatches per primer allowed with a no mismatch zone at the last two bases from 3’-end).

|  | NoCHL | Universal  Primer | Full-16S |
| --- | --- | --- | --- |
| Sequencing | MiSeq | NovaSeq | ONT |
| Alphaproteobacteria | 94.4 | 95.4 | 84.3 |
| Gammaproteobacteria | 94.4 | 94.2 | 81.1 |
| Cyanobacteriia | 5.6 | 92.8 | 81.5 |
| Planctomycetes | 91.3 | 94.7 | 79.8 |
| Verrucomicrobiae | 54.8 | 94.2 | 83.9 |
| Bacteroidia | 95.6 | 94.9 | 81.2 |
| Phycisphaerae | 65.8 | 96.3 | 78.5 |

Supplementary Table 3 | Percentage of identity between species present in the fungal mock community and fungal consensuses generated from ITS ONT sequences.

| Bacterial species | Consensus | Percent identity (%) | Query cover (%) |
| --- | --- | --- | --- |
| *Botrytinia fuckelian* | Consensus_12 | 98.53 | 100 |
| *Hypocreales* sp. LDF120R | Consensus_157 | 100 | 100 |
| *Hypocreales* sp. ANF130H | Consensus_157 | 100 | 100 |
| *Hypocreales* sp. LD40H | Consensus_157 | 99.25 | 75 |
| *Dentryphion penicillatum* ANF44R | Consensus_0 | 99.18 | 100 |
| *Dentryphion penicillatum* ANF44R | Consensus_2 | 94.10 | 97 |
| *Dentryphion penicillatum* ANF44R | Consensus_1483 | 92.77 | 96 |
| *Dentryphion penicillatum* ANF44R | Consensus_304 | 90.21 | 96 |
| *Dentryphion penicillatum* ANF44R | Consensus_41 | 95.95 | 73 |

Supplementary Table 4 | ASV tables with abundance, taxonomy, bootstrap and sequences for all sequencing methods. Data are filtered (i.e., read processing: dada2, chloroplast and homopolymers removal) and not normalized.
