# Supplementary figures and images for: A Practical Comparison of Short‐ and Long‐Read Metabarcoding Sequencing: Challenges and Solutions for Plastid Read Removal and Microbial Community Exploration of Seaweed Samples
Source: Mol Ecol Resour. 2025 Jun 4;25(7):e14129. doi: 10.1111/1755-0998.14129 (PMC12415815; doi:10.1111/1755-0998.14129)

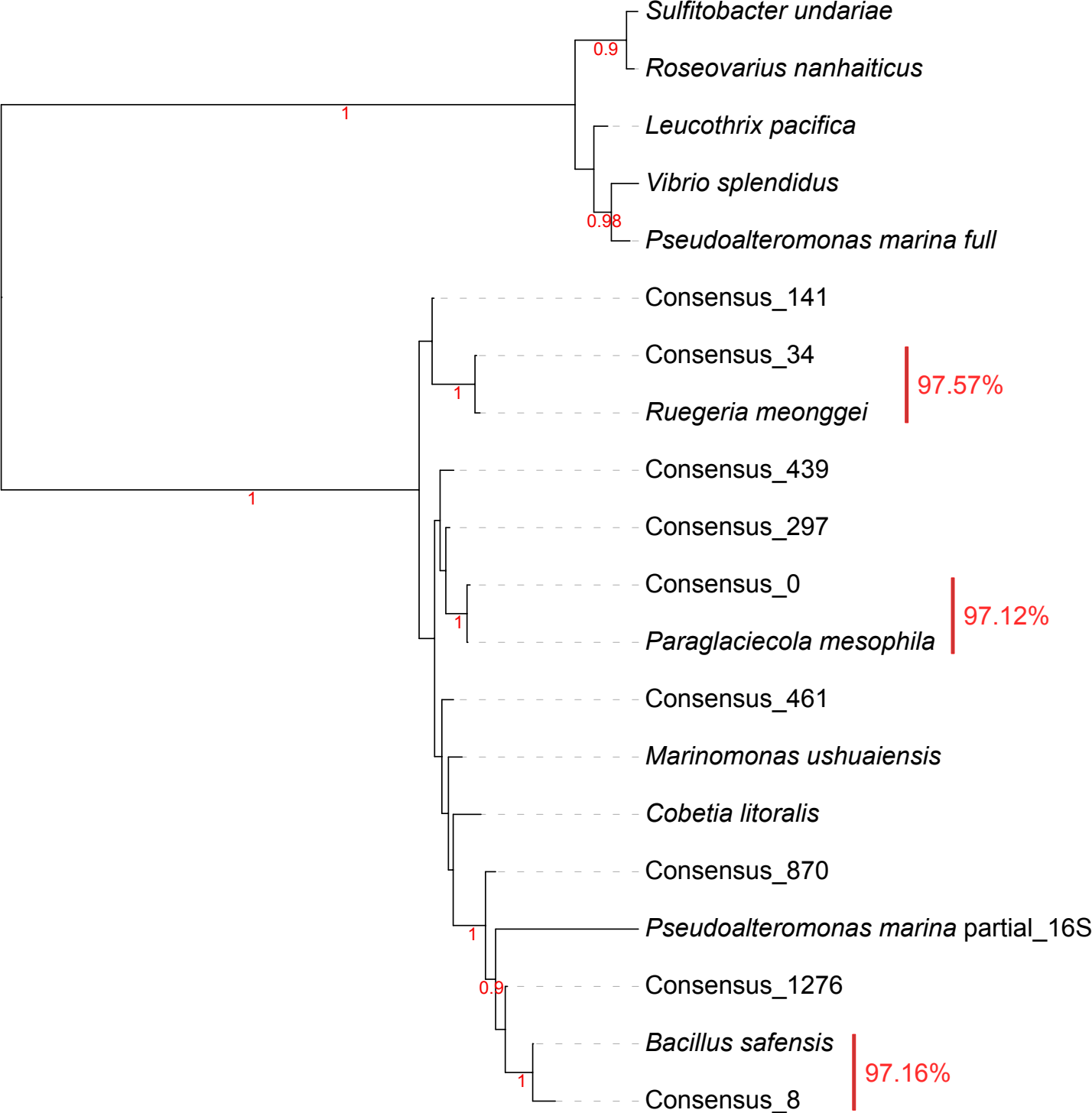

Supplement: Supplementary file 2 — Appendix S2 [file MEN-25-e14129-s002.zip › S1_mock_bact_ont_ngspecies.pdf]

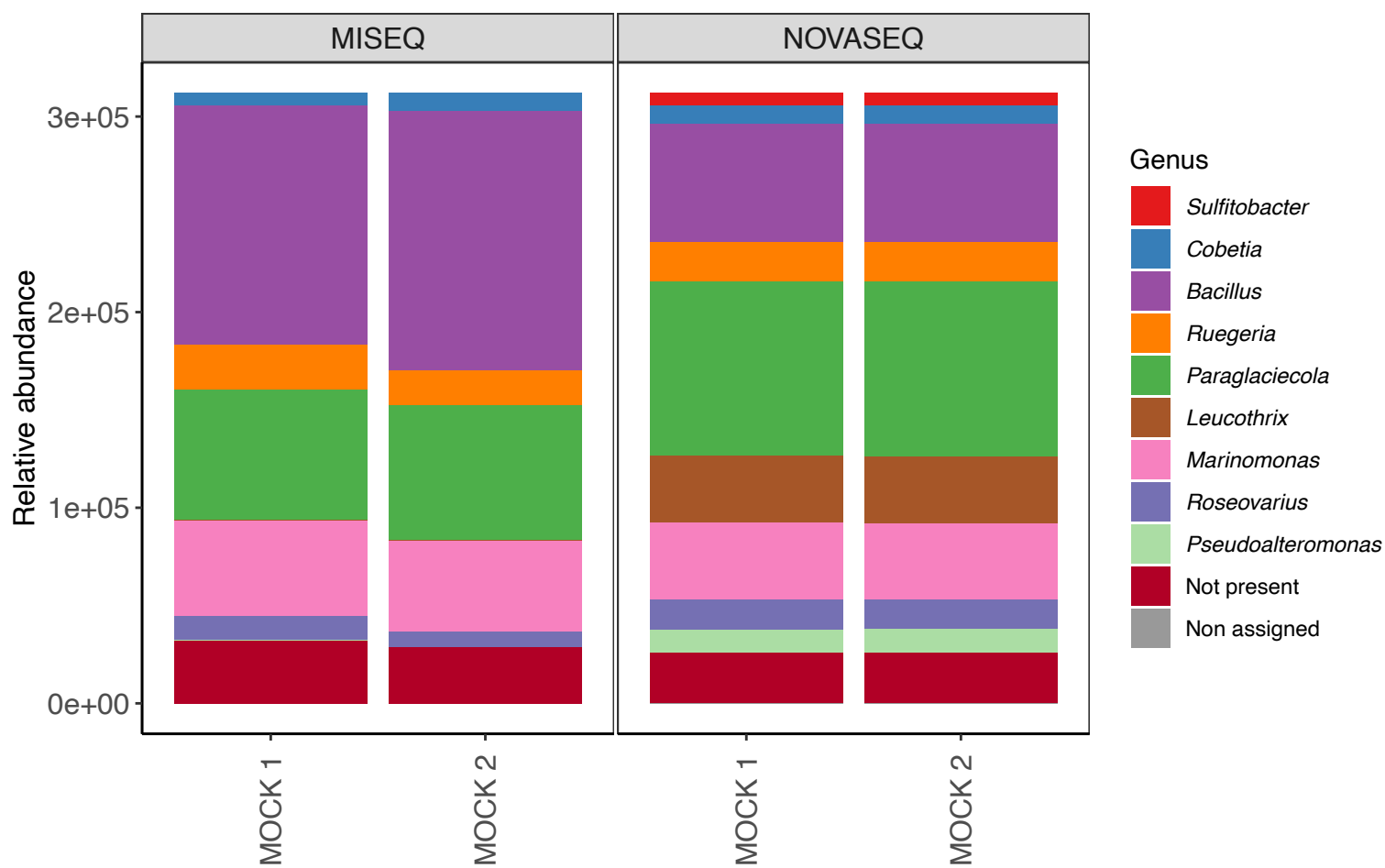

Supplement: Supplementary file 2 — Appendix S2 [file MEN-25-e14129-s002.zip › S2_mock_ms_ns.pdf]

A. SSU dataset

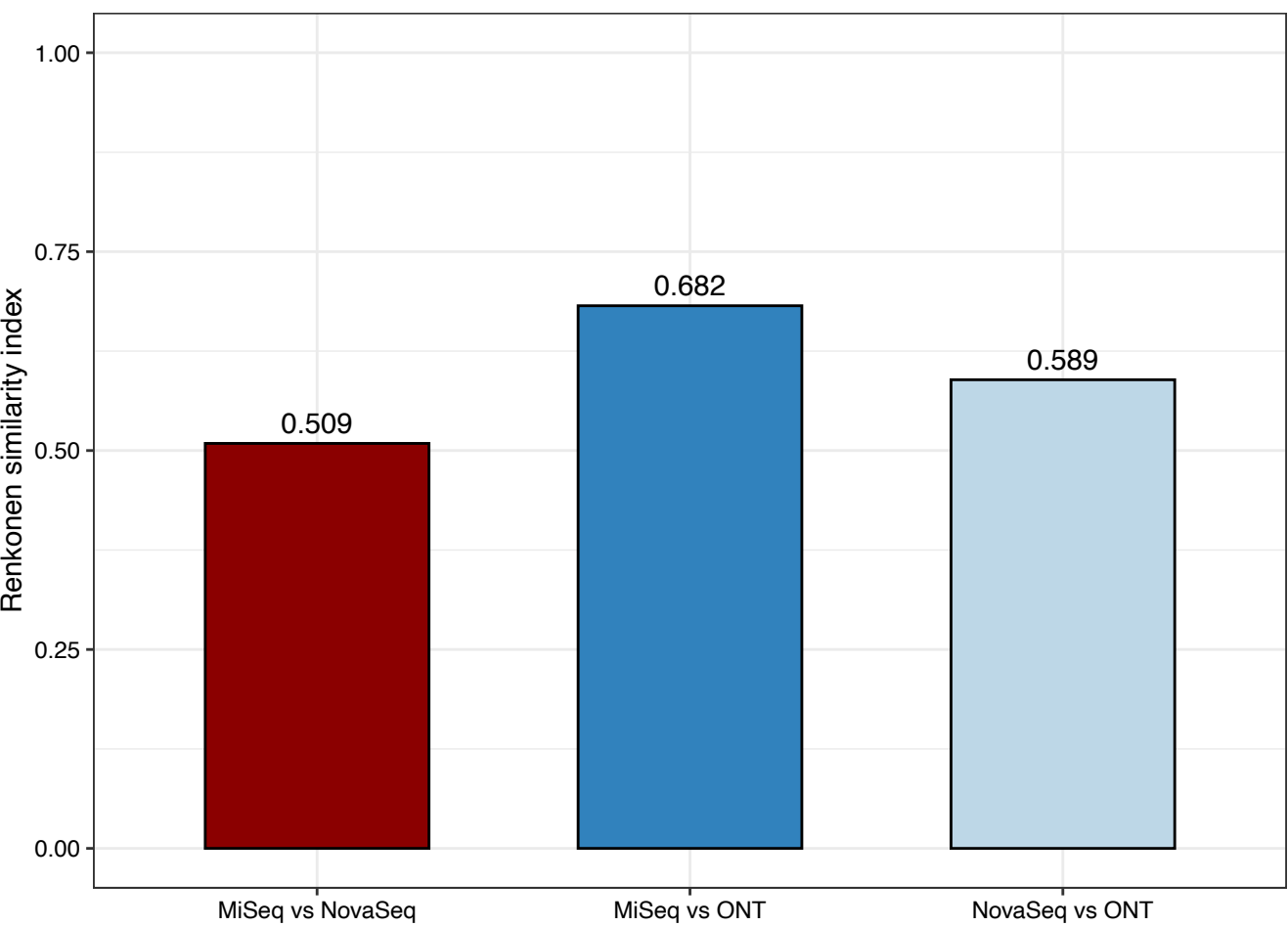

B. Fungal dataset

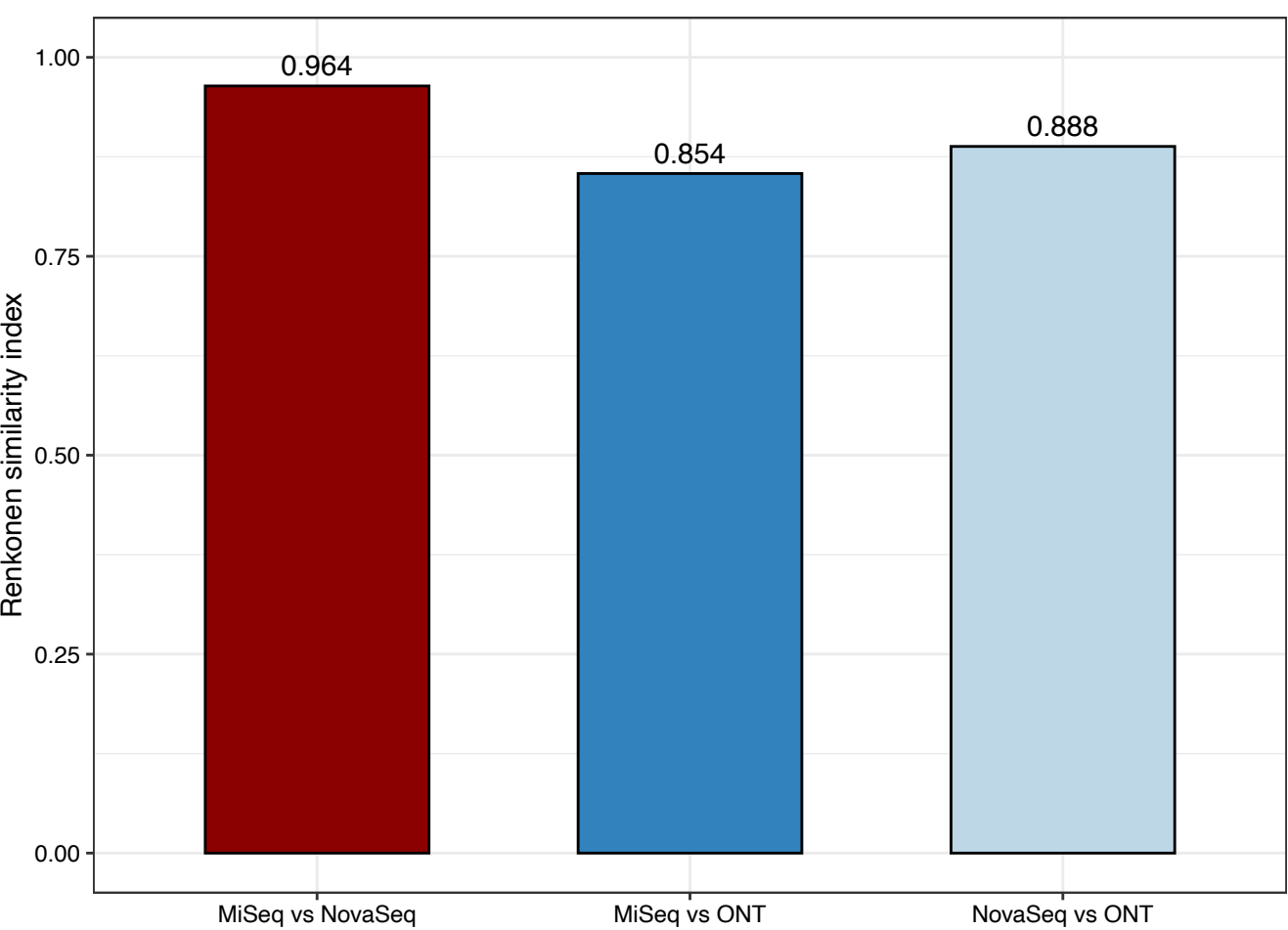

Supplement: Supplementary file 2 — Appendix S2 [file MEN-25-e14129-s002.zip › S3_Renkonen_similarity_index_combined.pdf]

A.

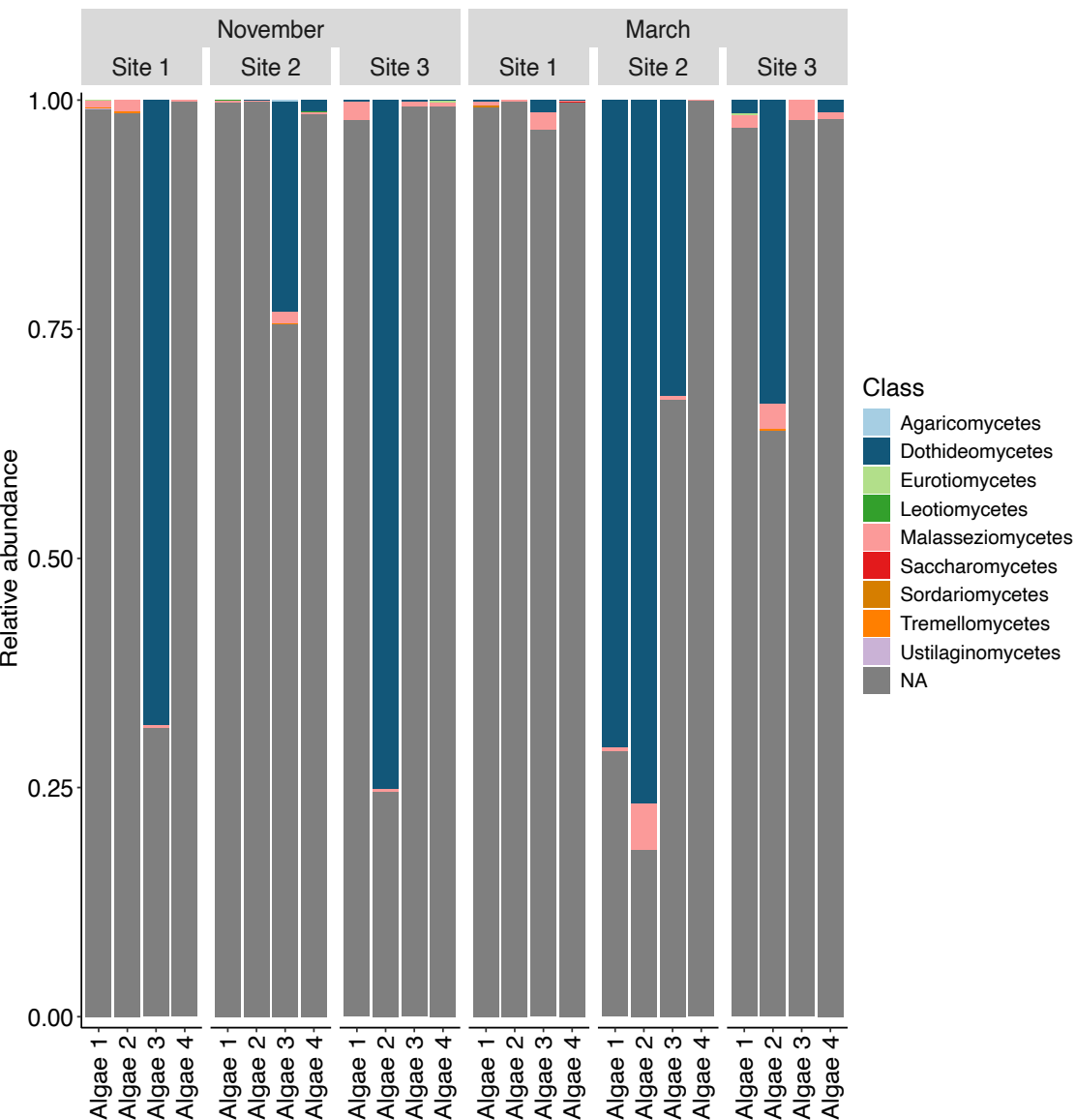

B.

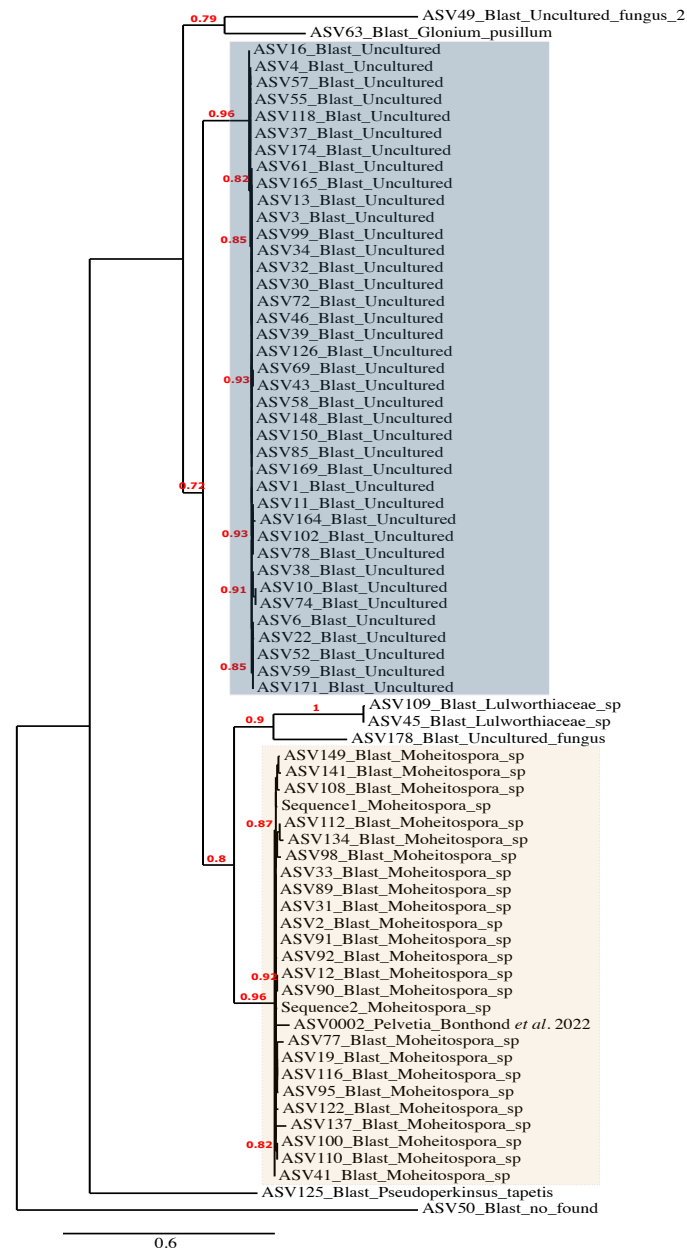

Supplement: Supplementary file 2 — Appendix S2 [file MEN-25-e14129-s002.zip › S4_combined_barplot_na_phylo_tree.pdf]
